# Longitudinal measurement invariance in prospective oral health-related quality of life assessment

**DOI:** 10.1186/s12955-016-0492-9

**Published:** 2016-06-07

**Authors:** Daniel R. Reissmann, Mike T. John, Leah Feuerstahler, Kazuyoshi Baba, Gyula Szabó, Asja Čelebić, Niels Waller

**Affiliations:** Department of Prosthetic Dentistry, Center for Dental and Oral Medicine, University Medical Center Hamburg-Eppendorf, Martinistrasse 52, 20246 Hamburg, Germany; Department of Diagnostic and Biological Sciences, University of Minnesota, Minneapolis, MN USA; Department of Psychology, University of Minnesota, Minneapolis, USA; Department of Prosthodontics, Showa University, Tokyo, Japan; Department of Prosthodontics, University of Pécs, Pécs, Hungary; Department of Prosthodontics, University of Zagreb and Clinical Hospital Centre, Zagreb, Croatia

**Keywords:** OHRQoL, OHIP, Measurement invariance, Response shift, Prospective studies, Longitudinal assessment

## Abstract

**Background:**

Prospective assessments of oral health-related quality of life (OHRQoL) changes are prone to response shift effects when patients reconceptualize, reprioritize, or recalibrate the perceived meanings of OHRQoL test items. If this occurs, OHRQoL measurements are not “invariant” and may reflect changes in problem profiles or perceptions of OHRQoL test items. This suggests that response shift effects must be measured and controlled to achieve valid prospective OHRQoL measurement. The aim of this study was to quantify response shift effects of Oral Health Impact Profile (OHIP) scores in prospective studies of prosthodontic patients.

**Methods:**

Data came from the Dimensions of Oral Health-Related Quality of Life Project. The final sample included 554 patients who completed the OHIP questionnaire on two occasions: pre- and post-treatment. Only items that compose the 14-item OHIP were analyzed. Structural equation models that included pre- and post-treatment latent factors of OHRQoL with different across-occasion constraints for factor loadings, intercepts, and residual variances were fit to the data using confirmatory factor analysis.

**Results:**

Data fit both the unconstrained model (RMSEA = .038, SRMR = .051, CFI = .92, TLI = .91) and the partially constrained model with freed residual variances (RMSEA = .037, SRMR = .064, CFI = .92, TLI = .92) well, meaning that the data are well approximated by a one-factor model at each occasion, and suggesting strong factorial across-occasion measurement invariance.

**Conclusions:**

The results provided cogent evidence for the absence of response shift in single factor OHIP models, indicating that longitudinal OHIP assessments of OHRQoL measure similar constructs across occasions.

**Electronic supplementary material:**

The online version of this article (doi:10.1186/s12955-016-0492-9) contains supplementary material, which is available to authorized users.

## Background

Oral health-related quality of life (OHRQoL) is an important patient-reported outcome in dentistry that characterizes the impact of oral diseases and dental treatments on quality of life. One of the most important tasks of an OHRQoL instrument is the measurement of change, that is, whether the patient’s situation has improved, stayed the same, or worsened. From a psychometric perspective, the measurement of change requires that a questionnaire measure the same construct (e.g., OHRQoL) on all occasions. Although this sounds simple, the relationships between questionnaire items and their underlying construct(s) may be complex. These relationships are typically characterized by a measurement model that need not stay constant across occasions. For instance, relative to a baseline, patients may change their internal standards of how they perceive OHRQoL when they are assessed at follow-up. In formal terms, a measurement model changes when, across measurement occasions, patients reconceptualize, reprioritize, or recalibrate the perceived meanings of test items [1]. Reconceptualization occurs when patients’ concepts of OHRQoL, as indicated by OHRQoL test items, changes across occasions. [2]. Reprioritization is defined as across-occasion variance in patient perceived importance of OHRQoL indicators. Finally, recalibration occurs when patients revise their internal standards of measurement. If any of these changes in the measurement model occurs, differences in perceived OHRQoL after treatment may reflect both changes in symptom profiles and changes in how patients perceive OHRQoL test items.

Measurement specialists have coined the term “response shift” [3] to characterize the psychometric consequences of the above phenomena. When present but not statistically controlled, response shift effects can sully the measurement of quality of life. This notion is of more than theoretical interest because response shift effects have been demonstrated in several medical [4–6] and dental studies [7–9]. Nevertheless, the presence of response shift effects in the oral health domain remains to be unambiguously established.

The Oral Health Impact Profile (OHIP) [10] is the most popular instrument for the assessment of OHRQoL. To improve measurement of change using the OHIP (and other OHRQoL instruments), response shift effects in prospective assessments need to be more accurately quantified to assess the true magnitude of dental intervention effects.

The aim of this study was to assess OHIP longitudinal measurement invariance by using structural equation models (SEM) to quantify response shift effects in pre- and post-treatment OHIP scores.

## Methods

### Subjects, study design, and setting

The data for this secondary data analysis came from the Dimensions of Oral Health-Related Quality of Life (DOQ) Project [11]. This project contains OHIP [10] data from general population subjects and prosthodontics patients from six countries (Croatia, Germany, Hungary, Slovenia, Sweden, Japan). For the present study, only baseline and follow-up data from dental patients from Croatia, Hungary, Germany, and Japan undergoing prosthodontic treatments were available for analysis. Data from prosthodontic patients in Sweden included data from the first assessment only [12, 13]. In Slovenia, patients received pre-treatment procedures for prosthodontic treatment (tooth pain was treated before more advanced dental therapy could be performed) [14]. Therefore, data from Sweden and Slovenia could not be used in the analyses. The included samples consisted of patients in university-based prosthodontic departments. All research was conducted in accordance with accepted ethical standards for research practice. Written informed consent was obtained from all participants prior to their enrollment. For further information regarding study characteristics, sampling, inclusion and exclusion criteria, and prosthodontic treatments performed within the included patient populations, see original publications [8, 15–18].

### Assessment of oral health-related quality of life

Oral health-related quality of life was assessed using validated, language-specific versions of the OHIP [19–23]. Each OHIP item describes a situation that impacts OHRQoL and asks subjects to rate how often they experienced a specific impact within the last month. Responses occur on a 5-point scale with higher numbers indicating greater impact: 0 = ‘never’, 1 = ‘hardly ever’, 2 = ‘occasionally’, 3 = ‘fairly often’, and 4 = ‘very often.’

Analyses were conducted on the widely used OHIP-14 short version [24]. OHIP-14 summary scores can range from 0 (no impact and best OHRQoL) to 56 (most impact and worst OHRQoL). In this manuscript, OHIP item numbers refer to the English-language 49-item OHIP version [10]. At baseline, Cronbach’s alpha [25] and the average inter-item correlations for the OHIP-14 data were .92 and .44, respectively. These values signal excellent reliability [26, 27] for this brief OHRQoL questionnaire.

Overall, the number of missing responses was small (less than 1 %) in the DOQ Project [11]. All OHIP-14 items were complete for 531 subjects (95.9 %) at baseline and for 538 subjects (97.1 %) at follow-up. Twenty-two subjects at baseline and twelve subjects at follow-up had one missing value, while two missing values were observed in one subject at baseline and four subjects at follow-up. Missing values were imputed using an individual’s median item response from the non-missing items of 49-item OHIP at each occasion.

Differences in OHIP-14 mean scores between baseline and follow-up were assessed using paired *t*-tests for the pooled study population and for each study separately.

### Establishing the measurement model

**Fig. 1:**
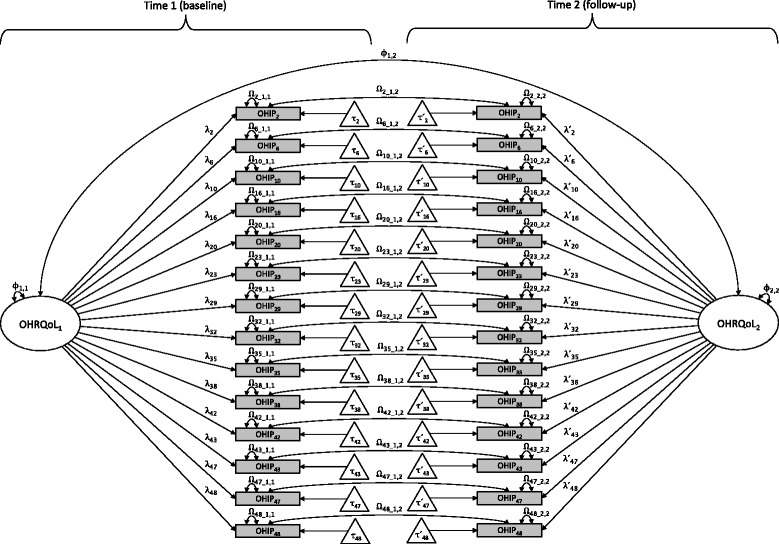
One-factor model for OHRQoL assessed with 14-item OHIP at two occasions; *Note*: rectangles represent items (i.e., measured or observed variables [OHIP_2_ – OHIP_48_]), ovals reflect latent common factors [OHRQoL_1_ and OHRQoL_2_], triangles indicate intercepts [τ_2_ − τ_48_ and τ*´*_2_ − τ*´*_48_], unidirectional arrows illustrate directional links (i.e., values of regression parameters for factor loadings [λ_2_ − λ_48_ and λ*´*_2_ − λ*´*_48_] and intercepts), and bidirectional arrows illustrate common factor variances [*Φ*_1,1_ and *Φ*_2,2_] and between-occasion factor covariance [*Φ*_1,2_] as well as item residual variances [ *Ω*_2_1,1_ − *Ω*_48_1,1_ and *Ω*_2_2,2_ − *Ω*_48_2,2_] and covariances [*Ω*_2_1,2_ − *Ω*_48_1,2_]. For clarity, notation is slightly different than that used in the text

To evaluate across-occasion measurement invariance for the OHIP-14, we fit a series of *a priori* defined confirmatory factor analysis (CFA) [28, 29] models and tested across-occasion measurement invariance following procedures outlined by Oort [30] and Gregorich [31]. Reconceptualization was evaluated by testing the dimensional and configural invariance of the measurement model. Reprioritization was assessed by testing metric invariance, and recalibration was evaluated by testing a model of strict factorial invariance. The CFA models included one common factor at each of the two assessment occasions because recent research suggests that, in many populations, OHIP item responses are well characterized by a single general factor [32, 33]. At each occasion, we used 14 occasion-specific OHIP items to identify a latent common factor. Additionally, we estimated across-occasion covariances among the latent factors and among the corresponding item residuals (Fig. 1).

The covariance structure among the 28 OHIP items (composed of the two sets of OHIP-14 items) was modeled as a two-factor confirmatory factor analysis (CFA).

1 $$ \Sigma ={\Gamma \Phi \Gamma}^T+\Omega $$

where Σ denotes the model-implied covariance matrix for the two sets of OHIP items; $$ \Gamma =\left(\begin{array}{cc}\hfill {\Gamma}_1\hfill & \hfill 0\hfill \\ {}\hfill 0\hfill & \hfill {\Gamma}_2\hfill \end{array}\right) $$ is a 28 × 2 matrix where Γ_1_ and Γ_2_ denote the occasion-specific factor loadings for the 14 OHIP items (subscripts refer to Time 1 and Time 2, respectively); $$ \Phi =\left(\begin{array}{cc}\hfill {\Phi}_{11}\hfill & \hfill {\Phi}_{12}\hfill \\ {}\hfill {\Phi}_{12}\hfill & \hfill {\Phi}_{22}\hfill \end{array}\right) $$ equals the variances and covariances among the common latent factors, where Φ_11_ and Φ_22_ represent the occasion-specific factor variances, and Φ_22_ represents the between-occasion factor covariance; and $$ \Omega =\left(\begin{array}{cc}\hfill {\Omega}_{11}\hfill & \hfill {\Omega}_{12}\hfill \\ {}\hfill {\Omega}_{12}\hfill & \hfill {\Omega}_{22}\hfill \end{array}\right) $$ denotes the item residual variances and covariances. Note that Φ_11_ and Φ_22_ are 14 × 14 diagonal matrices representing occasion-specific residual variances, and Φ_12_ is a diagonal matrix of across-occasion residual covariances. In our notation, diag(Ω_*kl*_) denotes the diagonal values of block matrix Ω_*kl*_ (*k* = {1,2}, *l* = {1,2})*.*

Item means were modeled by estimating item intercepts, *τ*, such that

2 $$ \mu \left(\boldsymbol{y}\right)=\tau +\Gamma \alpha $$

where $$ \mu \left(\boldsymbol{y}\right)=\left(\begin{array}{c}\hfill {\mu}_1\hfill \\ {}\hfill {\mu}_2\hfill \end{array}\right) $$ and *μ*_1_ and *μ*_2_ contain the occasion-specific observed item means; $$ \tau =\left(\begin{array}{c}\hfill {\tau}_1\hfill \\ {}\hfill {\tau}_2\hfill \end{array}\right) $$ and *τ*_1_ and *τ*_2_ contain the occasion-specific item intercepts; and $$ \alpha =\left(\begin{array}{c}\hfill {\alpha}_1\hfill \\ {}\hfill {\alpha}_2\hfill \end{array}\right) $$ is a 2 × 1 vector of latent factor means.

Due to the small number of OHIP response categories, the item residuals (i.e., the factor uniqueness scores that represent item variance not attributed to a common factor) are unlikely to be normally distributed. Thus it would be inappropriate to estimate the model parameters via maximum likelihood. For this reason, we fit competing CFA models with an unweighted least squares estimator using a mean and variance correction to calculate robust test statistics [34].

### Goodness-of-fit

To evaluate model fit, we used several goodness-of-fit indices recommended by Kline [29], including the log-likelihood chi-square test, the standardized root mean square residual (SRMR), the root mean square error of approximation (RMSEA), the comparative fit index (CFI), and the Tucker–Lewis index (TLI). Commonly applied guidelines [35] for adequate model fit suggest: SRMR: ≤ .08; RMSEA: ≤ .06; and CFI, TLI: ≥ .95. Accordingly, models not meeting these criteria were rejected.

### Model specifications for assessment of measurement invariance

In our first model, we tested whether the data could be characterized by single latent factors for each set of 14 OHIP items. If this model fails to be rejected, we have evidence for dimensional and configural invariance [31]. If the model is rejected, we have evidence for reconceptualization [30]. In Model 1, factor loadings (Γ_1_, Γ_2_), intercepts (*τ*_1_, *τ*_2_), and residual variances (diag(Ω_11_), diag(Ω_22_)) were freely estimated for each occasion. This unconstrained model includes the fewest number of parameter restrictions of the models under consideration. All elements of the factor covariance matrix, Φ, were freely estimated to allow the latent factor variances (i.e., the variances of the latent OHRQoL levels) to differ across occasions. For identification purposes, the first elements of Γ_1_ and Γ_2_ were fixed to 1.00, and the common latent factor means (*α*_1_ and *α*_2_) were fixed to 0.

Next, we fit a highly constrained model to test for response shifts effects in the across-occasion OHIP scores. In this model, we evaluated the presence of reprioritization and recalibration as operationalized by Oort [30]. In this framework, Γ_1_ ≠ Γ_2_ represents reprioritization, *τ*_1_ ≠ *τ*_2_ represents uniform recalibration, and diag(Ω_11_) ≠ diag(Ω_22_) represents non-uniform recalibration. For Model 2, all response shift parameters were constrained by specifying Γ_1_ = Γ_2_, *τ*_1_ = *τ*_2_, and diag(Ω_11_) = diag(Ω_22_), representing strict factorial invariance. Latent factor means were not constrained to be equal, *α*_1_ was fixed to 0, and *α*_2_ was freely estimated. Once again, to identify the model, the first elements of Γ_1_ and Γ_2_ were fixed to 1.00. To test for strict factorial invariance, we compared the relative model fit of the unconstrained Model 1 with the constrained Model 2, and tested for statistical significance using chi-square difference tests that were computed using the formulas described in Satorra and Bentler [36] for robust, mean and variance scaled chi-squares.

Finally, we fit a third model, Model 3, that can be viewed as a compromise between the fully unconstrained structure of Model 1 and the highly constrained structure of Model 2. In this model, the residual variances were freely estimated (diag(Ω_11_) ≠ diag(Ω_22_)) to allow for occasion-specific differences in item reliabilities. Once again, for identification purposes, the first elements of Γ_1_ and Γ_2_ were fixed to 1.00, and *α*_1_ was fixed to 0.

### Occasion-specific changes in OHRQoL

Effect sizes for across-occasion changes in OHRQoL were calculated for the 14 items and the latent factor means. Within the CFA framework outlined by Oort [30], across-occasion item mean differences are potentially composed of two components: true changes due to latent factor mean differences and changes due to response shifts. Because Model 3 includes no response shifts due to intercept or loading differences, the observed item changes equal the true item changes. Let

3 $$ \widehat{\Sigma}=\widehat{\Gamma}\widehat{\Phi}{\widehat{\Gamma}}^T+\widehat{\Omega} $$

denote the estimated parameters of EQ(1) and let ${\widehat{\sigma}}_{jk}$ be the row *j*, column *k* element of $\widehat{\Sigma}$ (i.e., the reproduced covariance matrix for the 28 OHIP items) such that ${\widehat{\sigma}}_{ii}$ denotes the estimated variance for item *i*(*i* = 1, …, 28). Given the parameter estimates in EQ(3), the *i*^*th*^ (*i* = 1, …, 14) true item-change effect size equals $\left({\mu}_{1(i)}-{\mu}_{2(i)}\right)/\sqrt{{\widehat{\sigma}}_{ii}+{\widehat{\sigma}}_{\left(i+14\right)\left(i+14\right)}-2{\widehat{\sigma}}_{\left(i+14\right)i}}$, where *μ*_1(*i*)_ denotes the *i*^*th*^ item mean at Time 1 and *μ*_2(*i*)_ denotes the associated mean at Time 2. Finally, the estimated latent factor effect size equals $\left({\widehat{\alpha}}_2-{\widehat{\alpha}}_1\right)/\sqrt{{\widehat{\Phi}}_{11}}$. A nonparametric bootstrap, using 10,000 samples, yielded 95 % effect size confidence intervals (CIs).

The latent change effect size for the factor means was compared to the effect size for the OHIP-14 summary scores. According to Cohen [37], an effect size of *d* = .2 is small, .5 is medium, and .8 is large. See the Additional file 1 for additional analyses and results regarding item-level reliability.

Computations were performed with STATA [38] and R [39]. All structural equation models were fit using the lavaan package [40] for R. Statistical significance was based on two-sided tests with Type I error rates set at .05 without adjustments for multiple comparisons.

## Results

### Characteristics of participants

A total of 554 prosthodontic patients with valid data for baseline (Time 1) and follow-up (Time 2) assessments were included in our analyses (Table 1). Mean OHIP summary scores decreased significantly from Time 1 to Time 2 in all study-specific samples (all *p* < .05; Table 1), corresponding to an increase in OHRQoL following prosthodontic treatment. Furthermore, most standard deviations (SDs) were lower at Time 2 than at Time 1, indicating lower score variability at follow-up. Consistent with these findings, all OHIP-14 item means and SDs decreased from Time 1 to Time 2 (Table 2).

**Table 1:** Demographic characteristics and OHRQoL change from Time 1 (baseline) to Time 2 (follow-up) of study participants

	N	Age [yrs]	Female	OHIP-14 sum score		
				Time 1	Time 2	
		*Mean (SD)*	*n (%)*	*Mean (SD)*		P-value*
All	554	55.3 (15.3)	286 (51.6)	10.5 (9.9)	7.2 (8.0)	< .001
Included samples						
Hungary [15]	62	54.9 (14.6)	37 (59.7)	13.2 (10.7)	6.8 (10.9)	< .001
Germany [16]	208	55.7 (15.8)	98 (47.1)	8.0 (7.3)	7.0 (6.8)	.005
Germany [17]	101	55.9 (14.6)	56 (55.4)	11.2 (10.6)	5.7 (8.4)	< .001
Germany [8]	123	54.6 (15.8)	61 (49.6)	8.0 (8.8)	6.6 (7.7)	.009
Japan [18]	30	60.8 (14.4)	23 (76.7)	16.0 (10.3)	10.8 (8.8)	< .001
Croatia (not published yet)	30	48.1 (12.7)	11 (36.7)	25.4 (8.1)	12.6 (4.8)	< .001

*Paired *t* test

**Table 2:** OHIP-14 item content and item means with standard deviations at Time 1 (baseline) and Time 2 (follow-up) based on ordinal 5-point response categories

Item #	Item content	Item mean (SD)	
		Time 1	Time 2
		*N* = 554	*N* = 554
Item 2	Trouble pronouncing words	0.73 (1.07)	0.55 (0.81)
Item 6	Taste worse	0.56 (0.89)	0.45 (0.74)
Item 10	Painful aching	1.05 (1.07)	0.82 (0.91)
Item 16	Uncomfortable to eat	1.19 (1.28)	0.86 (1.01)
Item 20	Self-conscious	1.07 (1.33)	0.57 (0.90)
Item 23	Tense	0.97 (1.12)	0.61 (0.85)
Item 29	Diet unsatisfactory	0.68 (1.08)	0.46 (0.78)
Item 32	Interrupt meals	0.71 (1.03)	0.50 (0.82)
Item 35	Difficult to relax	0.81 (1.09)	0.51 (0.80)
Item 38	Been embarrassed	0.84 (1.07)	0.48 (0.76)
Item 42	Irritable with others	0.44 (0.75)	0.34 (0.63)
Item 43	Difficulty doing jobs	0.43 (0.73)	0.32 (0.63)
Item 47	Life unsatisfying	0.78 (0.99)	0.48 (0.78)
Item 48	Unable to function	0.28 (0.62)	0.23 (0.55)

### Measurement models

Our initial SEM analysis supported Model 1 (Table 3) and suggested that the data were well characterized by a unidimensional model at each occasion. Thus we found support for configural invariance and no evidence for reconceptualization.

**Table 3:** SEM Model fit summary

Model	Specifications	Scaled *χ* ^2^	df	Scaled RMSEA	Scaled SRMR	Scaled CFI	Scaled TLI
# 1	Γ_1_ ≠ Γ_2_, *τ*_1_ ≠ *τ*_2_, Ω_11_ ≠ Ω_22_	606	335	.038	.051	.92	.91
# 2	Γ_1_ = Γ_2_, *τ*_1_ = *τ*_2_, Ω_11_ = Ω_22_	816	375	.046	.078	.87	.87
# 3	Γ_1_ = Γ_2_, *τ*_1_ = *τ*_2_, Ω_11_ ≠ Ω_22_	633	361	.037	.064	.92	.92

RMSEA - root mean square error of approximation; SRMR - standardized root mean square residual; CFI - comparative fit index; TLI - Tucker–Lewis index

Fit statistics for Model 2 indicated that this model was not a viable structural candidate for the data as the additional model constraints resulted in significantly poorer model fit compared to Model 1 (*χ*^2^(40) = 267, *p* <.01). Accordingly, a model enforcing strict factorial invariance and no response shift effects was not supported.

Model 3 fit considerably better than Model 2 (*χ*^2^(14) = 246, *p* <.01) but less well than Model 1 (*χ*^2^(26) = 84, *p* <.01). Notice, however, that according to our suite of fit indices, there are trivial differences between Model 1 and the more parsimonious Model 3. For these reasons, we retained Model 3 as the most parsimonious and interpretable structure for the 2-occasion OHIP data. The final parameter estimates for Model 3 are shown in Table 4. As expected, item residual variances were lower for Time 2 (diag(Ω_22_)) than for Time 1 (diag(Ω_11_)). Whereas there was no evidence for the presence of reprioritization and uniform recalibration, changes in residual variances suggested non-uniform recalibration in the measurement model.

**Table 4:** Parameter estimates for final model (# 3) and effect sizes of observed changes based on ordinal 5-point response categories and of true changes when item means were modeled by specifying a vector of model intercepts in final CFA model

Item #	Parameter estimates^a^					Effect sizes	
	*Γ*_1_ = *Γ*_2_	*τ*_1_ = *τ*_2_	*diag*(*Ω*_12_)	*diag*(*Ω*_11_)	*diag*(*Ω*_22_)	Observed item changes	True item changes
Item 2	1.000	0.762	.155	.701	.365	−.18	−.24
Item 6	0.731	0.594	.217	.565	.403	−.14	−.22
Item 10	0.872	1.042	.172	.816	.618	−.21	−.19
Item 16	1.439	1.202	.085	.731	.438	−.26	−.28
Item 20	1.394	0.993	.072	.903	.257	−.41	−.28
Item 23	1.236	0.942	.023	.570	.295	−.33	−.27
Item 29	1.188	0.719	.119	.550	.206	−.23	−.31
Item 32	1.047	0.735	.048	.579	.358	−.20	−.24
Item 35	1.010	0.784	.168	.736	.351	−.29	−.25
Item 38	1.219	0.811	.005	.484	.160	−.35	−.30
Item 42	0.752	0.480	.088	.308	.233	−.14	−.26
Item 43	0.787	0.471	.058	.265	.227	−.14	−.26
Item 47	1.064	0.760	.140	.490	.287	−.34	−.29
Item 48	0.600	0.332	.092	.222	.203	−.09	−.25
						OHIP-14 sum score	Latent factor mean
	*α*_1_	*α*_2_	*Φ*_12_	*Φ*_11_	*Φ*_22_	Observed change	True change
	.000	−.246	.229	.441	.284	−.34	−.37

^a^Subscripts refer to Time 1 and Time 2, respectively

*Γ*: factor loadings; τ: item intercepts; *Ω*: across-occasion and occasion-specific residual variances; α: latent factor means; *Φ*: variances and covariances among the common latent factors

Note: For the factor loadings (*Γ*), standard errors were ≤ .095. For the intercepts (τ), standard errors were ≤ .049. For the residual covariances (diag(Ω_12_)), standard errors were ≤ .039. For the residual variances at Time 1 (diag(Ω_11_)), standard errors were ≤ .067; at time 2 (diag(Ω_22_)), standard errors were ≤ .054. The standard error of *α*_2_ equals .029; the standard errors of *Φ*_12_, *Φ*_11_, and *Φ*_22_ equal .033, .058, and .042, respectively

### Observed and true changes in OHRQoL

As shown in Table 4, effect sizes for the observed item changes ranged from -.09 (Item 48) to -.41 (Item 20) and the effect sizes for the true item changes ranged from -.19 (Item 10) to -.31 (Item 29). Although the observed and true item effect sizes differed, the differences were generally small with no discernable pattern.

The effect size of the latent common factor change was -.37 (95 % CI: -.43 to -.31). This estimate suggests that the average Time 2 common factor score was .37 standard deviations lower than the average Time 1 common factor score. The effect size of the average OHIP-14 summary score was -.34 (95 % CI: -.42 to -.26), and not substantially different than the effect size of the latent factor.

## Discussion

Longitudinal measurement invariance of the OHIP was assessed with SEM to elucidate potential changes in across-occasion measurement models of OHRQoL. Data were well characterized by a model that included occasion-specific, single factor OHRQoL dimensions. On the basis of several goodness of fit statistics and model parsimony considerations, the data supported a model that specified across-occasion measurement invariance of the OHIP-14 latent structure. Hence, the results of this international study of OHRQoL suggest that the biasing effects of response shift [30] on OHIP scores is minimal.

As a measure of OHRQoL, the OHIP putatively reflects the theoretical structure of patient-perceived oral health across populations and different occasions. In the presence of response shift, changes in OHIP scores would not only represent true changes in the underlying OHRQoL construct. Rather, such observed changes would reflect changes in the measurement models. Because OHRQoL is a dynamic construct [41], the measurement model for this construct may change over time. However, the only change in the retained measurement model of the present study was in the item residual variances, that is, in the parts of the item variances that could not be attributed to the occasion-specific OHRQoL common factor. According to Oort’s [30] model this result reflects non-uniform recalibration. However, since this is a prospective cohort study with prosthodontic treatment between assessments, across-occasion changes in item residual variances seem not to be indicative of non-uniform recalibration. Specifically, because item means and SDs decrease from baseline to follow-up as an effect of treatment, residuals variances should also decrease as the item means approach their lower bounds. When treatment is maximally effective, all problems disappear, resulting in items means and variances of zero. Consequently, residual variances should also approach zero under ideal conditions of clinical improvement. Hence reduced item residual variances at Time 2 were expected due to post-treatment reduction in the number of oral health problems. Thus, our findings provide no evidence for significant response shift effects in prospective OHRQoL assessments using the OHIP in prosthodontic patients.

To our knowledge, this is the first study to apply SEM to response shift measurement in prospective OHRQoL assessments using the OHIP. Hence our ability to compare our findings with those in the existing literature is limited. Previous studies in dentistry have consistently reported response shift effects in the assessment of change scores [7–9]. All of these studies were prospective intervention studies with various types of prosthodontic treatments performed between baseline and follow-up. A general finding from this body of work is that treatment effects were larger when response shift was taken into account. Furthermore, several medical studies also demonstrated response shift effects with larger changes in health-related quality of life when considering response shift [4, 5]. This is in contrast to findings of no substantial response shift effects in the present study. Since different methods exist to detect response shift in patient-reported measures [2], inconsistencies among findings might be due to study design (prospective or retrospective). Furthermore, it is assumed that the occurrence of response shift depends on the presence of a catalyst [6], with medic al treatment being an important example. When no potential catalyst is present, that is, in individuals with chronic conditions who are in stable health, no substantial response shift effects exist [42]. Even though all patients in the present study received prosthodontic treatments that substantially improved their perceived oral health, this treatment-induced change in oral health might not have been large enough to catalyze changes in patients’ internal standards. This does not necessarily mean that prosthodontic treatment is not a catalyst in this context, but our data provide evidence that its effect on OHIP scores in terms of response shift is not clinically relevant.

This study has strengths and limitations. We applied state of the art CFA models to assess measurement invariance in prospective OHRQoL assessment. Although these methods have not been applied in dentistry often, they are well established in other medical fields [30] and in psychometrics [31]. The most commonly used approach to test for response shift or measurement invariance is the then-test method [2], which requires that the patients retrospectively rate their QoL at baseline from the perspective at follow-up. In contrast to the then-test method, SEM does not require multiple assessments at each occasion. Other advantages of our approach over the then-test is that our results are not susceptible to recall bias [4, 43] or to confounders that are attributable to “implicit theory of change” or “cognitive dissonance theory” [44, 45]. Although we cannot completely rule out these confounders, any confounding effects should be low or negligible due to the large time periods between baseline and follow-up assessments. For example, in one of the included studies [8], the between assessment time intervals averaged four months. Accordingly, baseline status should have no meaningful impact on follow-up information in a prospective assessment. When using SEM, we were able to quantify the stability or robustness of the theoretical structure of patient-perceived oral health across occasions. Using this approach, as opposed to the then-test, we were also able to evaluate the critically important property of across-occasion measurement invariance. Although we used only data from two occasions in the included studies, our findings should generalize to longitudinal studies with three or more assessments when no potential catalyst is present between assessments.

As noted earlier, our SEM analyses provided cogent evidence that OHIP-14 scores are well-characterized by a unidimensional measurement model. Given this result, we could not test for configural invariance separately from dimensional invariance. However, the one-factorial structure of OHRQoL assessed with OHIP has been corroborated in previous EFA and CFA analyses [32, 33], and our data fit the unconstrained single factor model for each occasion very well. Thus, our findings support both dimensional (same number of common factors) and configural invariance (common factors associated with identical items) for the OHIP short form. We used OHIP-14 as this is one of the most commonly applied OHRQoL questionnaires, with sufficient psychometric properties and less administrative burden than the longer versions [24, 46–49], making our findings relevant for most OHIP research.

This study used pooled data from several international studies to create stable models with precise parameter estimates. The included samples consisted of patients in university-based prosthodontic departments and did not differ substantially in age, gender, or perceived improvements in OHRQoL following prosthodontic treatment. Furthermore, we found no signs that cross-cultural measurement invariance was violated, which is in line with a previous study in a similar setting [50]. Because patients in this study were typical dental patients [11], our findings should generalize well to other dental patient populations.

## Conclusions

In conclusion, this study clearly demonstrated that patients’ observed changes in perceived oral health are not confounded by response shift effects in the measurement of OHRQoL using the OHIP-14. In other words, changes in OHIP-14 mean scores due to treatment can be trusted to reflect true change in patients’ OHRQoL.

## Abbreviations

CFA, Confirmatory factor analysis; CFI, Comparative fit index; DOQ, Dimensions of Oral Health-Related Quality of Life; OHIP, Oral Health Impact Profile; OHRQoL, Oral health-related quality of life; RMSEA, Root mean square error of approximation; SEM, Structural equation model; SRMR, Standardized root mean square residual; TLI, Tucker–Lewis index.

## Additional file

Additional file 1: Item-level reliability. (DOCX 101 kb)
