# Supplementary material for: Longitudinal measurement invariance in prospective oral health-related quality of life assessment
Source: Health Qual Life Outcomes. 2016 Jun 7;14:88. doi: 10.1186/s12955-016-0492-9 (PMC4897855; doi:10.1186/s12955-016-0492-9)
Supplement: Additional file 1: — Item-level reliability. (DOCX 101 kb) [file 12955_2016_492_MOESM1_ESM.docx]

Longitudinal measurement invariance in prospective oral health-related quality of life assessment

**Daniel R. Reissmann**^1,2^ (Corresponding author)
d.reissmann@uke.de

**Mike T. John**^2^johnx055@umn.edu

**Leah Feuerstahler**^3^
feuer024@umn.edu

**Kazuyoshi Baba**^4^
kazuyoshi@dent.showa-u.ac.jp

**Gyula Szabó**^5^gyula.szabo@aok.pte.hu

**Asja Čelebić**^6^celebic@sfzg.hr

**Niels Waller**^3^nwaller@umn.edu

^1^ Department of Prosthetic Dentistry, Center for Dental and Oral Medicine, University Medical Center Hamburg-Eppendorf, Martinistrasse 52, 20246 Hamburg, Germany

^2^ Department of Diagnostic and Biological Sciences, University of Minnesota, Minneapolis MN, USA

^3^ Department of Psychology, University of Minnesota, Minneapolis, USA

^4^ Department of Prosthodontics, Showa University, Tokyo, Japan

^5^ Department of Prosthodontics, University of Pécs, Pécs, Hungary

^6^ Department of Prosthodontics, University of Zagreb and Clinical Hospital Centre, Zagreb, Croatia

**Appendix**

***Item-level reliability***

In this section we demonstrate how CFA parameters can be used to estimate occasion-specific item-level reliabilities for the OHIP-14. From our previously described model (see EQ(1) *supra*),

$\text{var}\left( y_{i} \right)=\sigma_{ii}=\Gamma_{i\cdot}\Phi\Gamma_{i\cdot}^{T}+\Omega_{ii}$,

where $\Gamma_{i\cdot}$ $\left( i=1,\ldots,28 \right)$denotes the $i$^th^ row of $\Gamma$ (expressed as a row vector) and all other terms are as previously defined. In this model, the observed item variance is decomposed into two components: a variance component that is associated with the common factors ($\Gamma_{i\cdot}\Phi\Gamma_{i\cdot}^{T}$) and a residual variance component ($\Omega_{ii}$). Note that from standard psychometric theory (Kline 2011), the residual variance can be further bifurcated into components due to reliable and unreliable variance. In many models, separate estimates of these sources of variance are unattainable. However, as we now demonstrate, these estimates are attainable in the response shift models under study. Estimated item reliabilities can be computed as follows.

Let

$\Omega_{ii}=s_{i}^{2}+e_{i}^{2}$,

where, for item $i$ ($i=1,\ldots,14)$, denotes the reliable component of the residual variance and $e_{i}^{2}$ denotes the unreliable component of the residual variance. Furthermore, to model the Time 2 residual variances, let

$\Omega_{(i+14)(i+14)}=s_{i+14}^{2}+e_{i+14}^{2}$.

Assume that

$s_{i}^{2}=s_{i+14}^{2}$.

That is, we assume that, for all items, the specific variance components are equal across occasions (but not across items). According to the assumptions of our model and the Cauchy-Schwartz inequality,

$\Omega_{i\left( i+14 \right)}\leq s_{i}^{2}=s_{i+14}^{2}$.

Formally defined, a reliability index equals the ratio of reliable variance to total variance. Thus, under the above assumptions, the reliability of item $i$, ($i=1,\ldots,14)$––that is, the Time 1 item reliabilities––must be greater than or equal to

|  | $(\Gamma_{i\cdot}{\Phi\Gamma_{i\cdot}^{T}+\Omega_{i\left( i+14 \right)})}/\left( \Gamma_{i\cdot}\Phi\Gamma_{i\cdot}^{T}+\Omega_{ii} \right),$ | (4) |
| --- | --- | --- |

and the reliability of item $(i+14)$, ($i=1,\ldots,14)$––that is, the Time 2 item reliabilities––must be greater than or equal to

|  | $(\Gamma_{\left( i+14 \right)\cdot}{\Phi\Gamma}_{\left( i+14 \right)\cdot}^{T}+\Omega_{i(i+14)})/(\Gamma_{\left( i+14 \right)\cdot}\Phi\Gamma_{\left( i+14 \right)\cdot}^{T}+\Omega_{(i+14)(i+14)}).$ | (5) |
| --- | --- | --- |

Table 1 reports the occasion-specific OHIP-14 item reliabilities computed using the above formulae and the parameter estimates from Model 3. Notice that, for all items and occasions, these item reliabilities are impressively large. Moreover, for most items, the item reliabilities increase from Time 1 to Time 2. This suggests that the OHIP-14 can be used to generate reliable symptom information as well as reliable information about the underlying OHRQoL construct.

Table 1 – Estimated OHIP-14 item-level reliabilities

| **Item #** | **Reliability** | |
| --- | --- | --- |
|  | Time 1 | Time 2 |
| Item 2 | .521 | .676 |
| Item 6 | .566 | .666 |
| Item 10 | .440 | .465 |
| Item 16 | .607 | .656 |
| Item 20 | .527 | .771 |
| Item 23 | .560 | .627 |
| Item 29 | .632 | .857 |
| Item 32 | .500 | .537 |
| Item 35 | .520 | .714 |
| Item 38 | .579 | .734 |
| Item 42 | .605 | .631 |
| Item 43 | .615 | .582 |
| Item 47 | .646 | .758 |
| Item 48 | .658 | .636 |
